# Introducing a secondary segmentation to construct a radiomics model for pulmonary tuberculosis cavities

**DOI:** 10.1007/s11547-023-01681-y

**Published:** 2023-07-20

**Authors:** Tamarisk du Plessis, Gopika Ramkilawon, William Ian Duncombe Rae, Tanita Botha, Neil Alexander Martinson, Sarah Alice Parry Dixon, Andre Kyme, Mike Michael Sathekge

**Affiliations:** 1 Department of Nuclear Medicine, Faculty of Health Sciences, University of Pretoria, Pretoria, South Africa; 2 Department of Statistics, Faculty of Natural and Agricultural Sciences, University of Pretoria, Pretoria, South Africa; 3 Medical Imaging Department, Prince of Wales Hospital, Sydney, Australia; 4 Perinatal HIV Research Unit (PHRU), University of the Witwatersrand, Johannesburg, South Africa; 5 Johns Hopkins University Centre for TB Research, Baltimore, MD USA; 6 School of Biomedical Engineering, University of Sydney, Sydney, Australia

**Keywords:** Radiomics, Segmentation, Pulmonary tuberculosis, Sliding window

## Abstract

**Purpose:**

Accurate segmentation (separating diseased portions of the lung from normal appearing lung) is a challenge in radiomic studies of non-neoplastic diseases, such as pulmonary tuberculosis (PTB). In this study, we developed a segmentation method, applicable to chest X-rays (CXR), that can eliminate the need for precise disease delineation, and that is effective for constructing radiomic models for automatic PTB cavity classification.

**Methods:**

This retrospective study used a dataset of 266 posteroanterior CXR of patients diagnosed with laboratory confirmed PTB. The lungs were segmented using a U-net-based in-house automatic segmentation model. A secondary segmentation was developed using a sliding window, superimposed on the primary lung segmentation. Pyradiomics was used for feature extraction from every window which increased the dimensionality of the data, but this allowed us to accurately capture the spread of the features across the lung. Two separate measures (standard-deviation and variance) were used to consolidate the features. Pearson’s correlation analysis (with a 0.8 cut-off value) was then applied for dimensionality reduction followed by the construction of Random Forest radiomic models.

**Results:**

Two almost identical radiomic signatures consisting of 10 texture features each (9 were the same plus 1 other feature) were identified using the two separate consolidation measures. Two well performing random forest models were constructed from these signatures. The standard-deviation model (AUC = 0.9444 (95% CI, 0.8762; 0.9814)) performed marginally better than the variance model (AUC = 0.9288 (95% CI, 0.9046; 0.9843)).

**Conclusion:**

The introduction of the secondary sliding window segmentation on CXR could eliminate the need for disease delineation in pulmonary radiomic studies, and it could improve the accuracy of CXR reporting currently regaining prominence as a high-volume screening tool as the developed radiomic models correctly classify cavities from normal CXR.

## Introduction

Tuberculosis (TB) is one of the top ten causes of death worldwide according to the World Health Organization [1]. However, an estimated 66 million lives were saved in the past two decades through TB diagnosis and treatment [1]. Early and accurate diagnosis is vital in fighting this global battle against TB spread and infections. Planar chest X-rays (CXR), in combination with biological methods, are commonly used to screen for or diagnose pulmonary TB (PTB) in patients at high risk of TB disease. CXR is the most widely accessible imaging modality in high TB burdened countries and is regaining prominence as a high-volume screening modality [2]. Advantages of CXR include that it is relatively inexpensive, fast, noninvasive and a good indicator of the extent of disease in the lungs [3]. Some disadvantages are that expert X-ray interpreters are often scarce in resource-limited countries [4], and results are influenced by intra-observer subjectivity [5]. To lower the subjectivity associated with X-ray interpretation, data science research has focussed on quantifying and analyzing features on CXR [6, 7].

Radiomic feature extraction is one such tool that can be used to quantify disease characteristics, or features, and assess progression from serial medical images in the same patient, as it makes use of statistically based imaging analysis algorithms to convert medical images into mineable high-dimensional data [8, 9]. Radiomics can extract relevant image information that can comprehensively assess the entire two-dimensional landscape in the region-of-interest (ROI) [10]. Radiomic libraries can extract hundreds to thousands of features per image. As an image mining tool generating such big data, radiomics naturally lends itself to the application of machine learning or deep learning approaches for developing signatures or advanced model building [10, 11].

Radiomics is a trending research technique in oncology imaging, but it is less studied in non-neoplastic pathologies such as PTB [12, 13]. A recent systematic review showed that radiomic feature extraction, for the purpose of PTB diagnosis or differentiation from other pulmonary pathology, has only been applied in five studies [12]. In all five studies, CT or PET/CT scans were used as the input imaging modality [12]. The review highlighted the need, and the challenges, of applying feature extraction to chest X-rays [12]. One challenge is that PTB has diverse radiological presentations: cavities, adenopathy, infiltrates and plural effusions, miliary pattern with the disease spreading across either a relatively small proportion of a single lung, or with extensive bilateral disease. Accurate segmentation of these diverse disease presentations is difficult and time consuming and not always feasible with large data sets [14] and can result in significant observer-bias [6]. This is a major limitation in the quantification of non-neoplastic diseases, because variability in segmentation is the biggest cause of irreproducible radiomics outcomes [10].

Several radiomic features are interpreted differently when subjected to inter- and intra-observer assessments in delimiting ROIs [14]. Some articles use manual segmentation by expert readers as the ground truth for segmentation [15], but both manual and semi-automatic segmentation have limitations, while fully automatic segmentation models are fast and have good reproducibility. Many segmentation algorithms have already been trained with deep-learning methods to perform automatic segmentation tasks for various imaging modalities, including CXR [14]. These models are used primarily for organ segmentation but cannot yet identify the pathology [16], especially in non-neoplastic pulmonary diseases. The principal aim of our study was to develop a segmentation method, applicable to chest X-rays that could eliminate the need for precise disease delineation in the lungs. This segmentation method will be applicable to any CXR quantification study, but we developed it specifically for radiomic feature extractions.

In recent years, radiomics has gained increasing popularity due to its ability to quantify medical images and for the construction of radiomic signatures, nomograms, machine learning classifiers and models to assist in disease diagnosis, prediction of disease status, response to treatment and disease prognosis [17]. Radiomics improve discrimination performance and detection of medical images compared with those made by radiologists alone [9, 17, 18]. Our study used radiomics to develop a model to automatically differentiate normal CXR from CXR with cavities, to assist clinicians with improved and faster PTB diagnosis.

PTB has many radiological presentations, but thick-walled cavities are generally an excellent radiological indicator of active PTB that with treatment and time resolve into thin-walled smooth cavities in treated TB [19]. These cavities cause textural changes in the lung that are visually apparent. Cavities were therefore selected as the radiological TB expression under investigation for this study.

## Methods

Every step in a the multi-step workflow of radiomics can influence the results and reproducibility [20]. To address this, the Imaging Biomarker Standardization Initiative (IBSI), published in 2019, aims to standardize image biomarker nomenclature and definitions to standardize image biomarkers extraction [21]. The IBSI guidelines were adhered to where possible during this study.

### Patient selection

This is a retrospective study that used a dataset consisting of 266 posteroanterior (PA) CXR of patients diagnosed with laboratory-confirmed PTB between August 2013 and July 2018. The CXR were radiologically reported on by clinicians who were part of the initial study. Additionally for this study, a single experienced TB clinician was asked to retrospectively review the X-rays individually, blinded to the previous reports. The second observer confirmed the CXR classifications as either normal (*n* = 71) or with the presence of cavities (*n* = 195). All CXR with discordant or indeterminate classifications were removed from this analysis. In this retrospective dataset, the acquisition equipment was not recorded, but it can be assumed that various imaging units were used.

### X-ray pre-processing

The original dataset included images in DICOM format which were acquired using non-standardized patient positions, image sizes, orientations, photometric interpretations and bit depths using a range of different imaging units. Total Image Converter version 8.2.0.237 (by CoolUtils.com file converters) was used for initial pre-processing to ensure a uniform dataset. The following pre-processing steps were applied: Manually cropped all images to square dimensions, corrected unconventional photometric interpretations on some images and converted DICOM images to PNG format. Python version 3.7.6 was used to interpolate all images to 256 × 256 pixels with bilinear interpolation and to convert the conventional RGB type for PNG format to scalar type as required by the radiomics library.

### Primary image segmentation

**Fig. 1:**
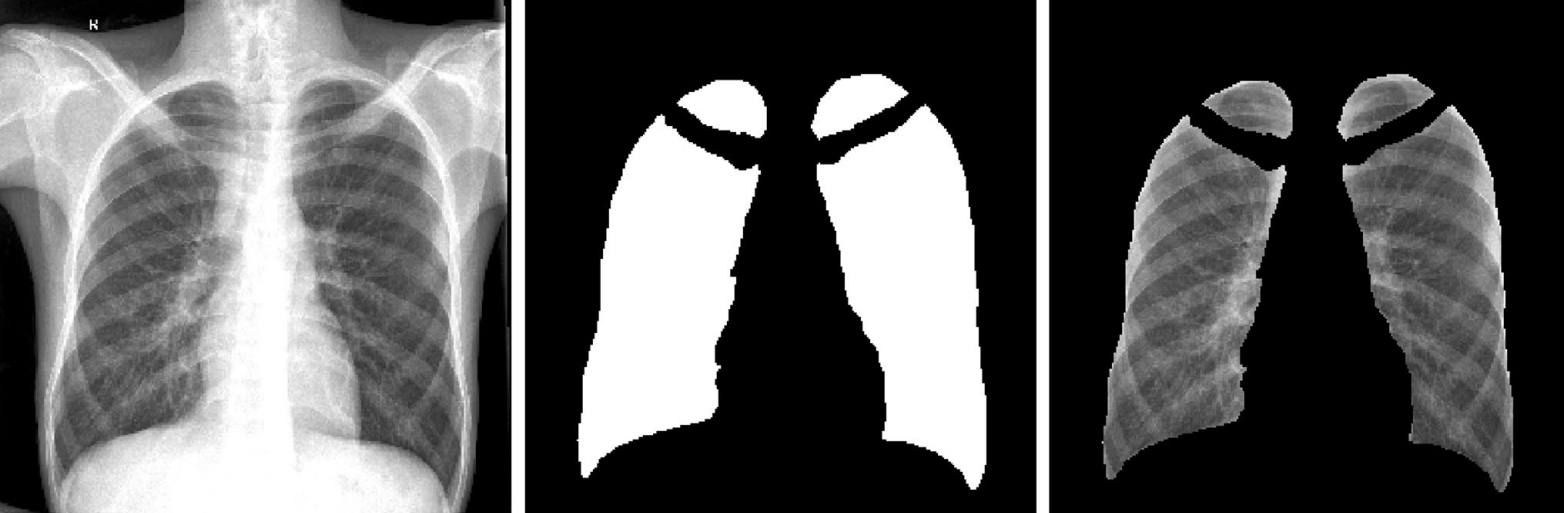
Output of the segmentation model (from left to right): The original image, the mask output (multiplied by 255 to be visible) and the mask superimposed with the image that was used to evaluate the segmentation accuracy

A fully automatic in-house segmentation model was used to segment the lung fields [22]. This U-net-based model was trained and validated on a publicly available Chest X-ray 14 Dataset (CX14) [22]. It was then tested on an unseen publicly available dataset, the JSRT dataset, and achieved a maximum Intersection over Union (IoU) of 0.8301, 0.9210, and 0.7791 for the heart, lungs and clavicles, respectively [22]. The segmentation model resizes images to 256 × 256 pixels with bilinear interpolation before segmenting the lungs as a 256 × 256 pixel mask output. Because all images had been previously interpolated to the same dimensions as the masks, they could simply be multiplied with the masks to visually evaluate the accuracy of the segmentation model on our unseen dataset (Fig. 1). All CXR were correctly segmented, and no manual corrections were needed.

### Secondary segmentation

To create the secondary sliding window segmentation a square mask of *n x n*, pixels was created and called the sampling window (*w*). This sampling window was selected to be large enough for the enclosed region to exhibit similar characteristics to those of the underlying region and at the same time to be as small as possible to enable the accurate detection of borders between adjacent textural regions. This window will slide over the image in both vertical and horizontal dimensions with a predetermined window step size (*w*_step_). *W*_step_ therefore determines the number of pixels that the sampling window slides across at each step and determines how well boundaries between features are resolved. There is a trade-off: if *w*_step_ is too large boundaries will be unresolved, while if *w*_step_ is too small then extended times are devoted to computation, and the window would place bounds on many of the features and increase their variability.

The sliding window masks were created in Python (version 3.7.6) using Numpy.array() and PIL.Image() functions. A square window (*w*) of 16 × 16 pixels and a window step size (*w*_step_) of 4 pixels was selected. The window will move, or slide, from one side of the CXR to the other in both *x*- and *y*-dimensions to create a window matrix of 61 × 61 windows. The number of windows in the matrix can be calculated using Eq. 1 where *[P*_*x*_*, P*_*y*_*]* is the dimensions of the window matrix, *[n*_*x*_*, n*_*y*_*]* is the dimensions of the image in pixels, *w* is the window size and *w*_step_ is the window step size.

1 $$P_{x} = \frac{{\left( {n_{x} - w} \right)}}{{w_{{{\text{step}}}} }} + 1 \quad {\text{and}}\quad P_{y} = \frac{{\left( {n_{y} - w} \right)}}{{w_{{{\text{step}}}} }} + 1$$

The sliding windows will therefore cause the effective dimensionality of each image’s features to increase by a factor 3721 (61 × 61). This resolution is adequate to resolve the change in the radiomic features across the lung, within an acceptable computational time.

### Radiomic feature extraction

**Fig. 2:**
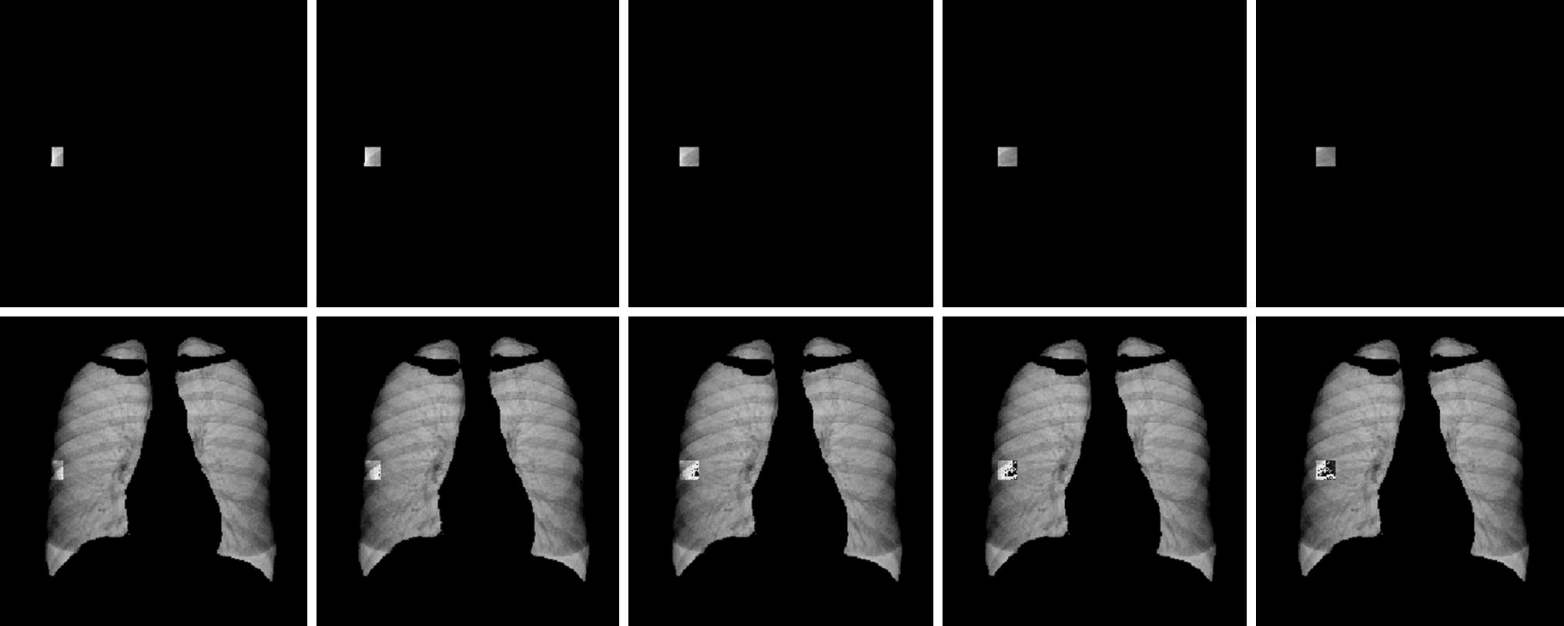
Above: Example of 5 sliding windows, sliding horizontally in the *y* axis (window coordinates [*P*_*x,*_* P*_*y*_] = [30, 9], [30, 10], [30, 11], [30, 12] and [30, 13]) superimposed on the lung mask and the CXR. Below: The same sliding windows, inverted to allow visualization of the lung mask

The sliding window masks were superimposed on the primary segmented lung mask of each CXR (see Fig. 2). Radiomic features were extracted from each window in the window matrix if the window was not masked off by the lung segmentation.

The Pyradiomics library (version 3.0) was used to extract 93 two-dimensional (2D) first-order and texture-features from each sliding-window on each CXR. Shape-based features will be meaningless for the purpose of this study, as these features use the masked ROI for calculating the values and was omitted.

### Dimensionality reduction

Statistical analysis was performed using R Software (version 4.1.3; http://www.r-project.org/). The secondary segmentation caused an approximately 3000-fold increase in the dimensionality of the features extracted. Before traditional dimensionality reduction methods could be applied, additional measures were introduced, namely standard deviation (SD) and variance, to quantify and capture the change in features over the lung region [23].

These two measures were calculated for each of the 93 features in the cavity dataset (195 CXR) and the normal dataset (71 CXR), respectively. Using the resultant expressions of the features in these two measures formed the first step in the feature selection process to limit the complications of dimensionality which arise from the over-abundance of features. For dimensionality reduction, Pearson’s correlation coefficient $\rho$ was used to identify the uncorrelated features [24, 25].

The Pearson correlation coefficients between 0 and 1 indicate a positive correlation, correlations equal to zero indicate no correlation and correlations between -1 and 0 indicate a negative correlation. These correlation coefficients were calculated for each feature pair. Feature pairs with absolute correlations ($|\rho |$) greater than a pre-determined cut-off value were removed. Three cut-off values were considered for this study, namely 0.7, 0.8 and 0.9, and later examined to decide which was the most appropriate for the purpose of dimensionality reduction. In addition to the removal of highly correlated features, only features common in both the cavity- and normal datasets were retained.

### Model development

To apply this developed radiomic signature in a meaningful manner, a random forest model was constructed to differentiate cavities seen on CXR of people suffering from PTB and normal CXR [26]. This random forest model was used due to its attractive computational features and classification performance as it is robust to overfitting data by design [26]. Due to the imbalance between the cavity and normal samples, a random walk oversampling technique was applied to improve the model’s performance [27, 28]. Four other sampling strategies were also considered and recorded in the results section. After adjustments to the data were made to ensure equal representation, the entire dataset was split into the training and testing sets with a 70/30% split, respectively.

Two separate random forest models were built, using the same CXR set on which the SD- and variance signatures were developed, respectively [29]. For both random forest models, a grid search cross-validation method was employed to determine the ideal number of variables to try at each tree split with 15-folds and 5 repeats to further limit overfitting. Across both random forest models, the grid search cross-validation indicated that the ideal number of variables to try at each tree split was 1. The performance of the models was validated using the testing set, AUC measure, accuracy, sensitivity, and specificity.

## Results

### Signature results

**Fig. 3:**
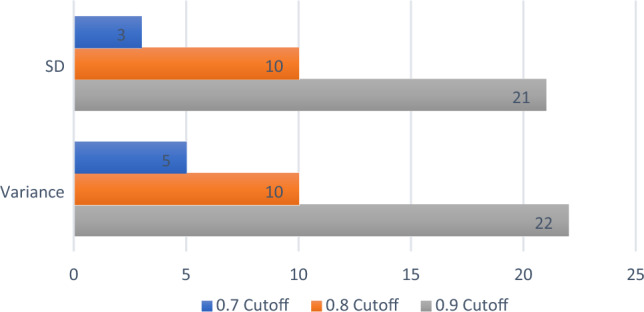
The number of common features retained for the two different consolidation measures when different cut-off values were considered in the Pearson’s correlation analysis

The first step in finding the signature was to determine the optimal cut-off value for the Pearson’s correlation. To evaluate this, only the number of features common to both the cavity- and normal datasets were considered. Figure 3 indicates the number of features retained for the two different consolidation measures when different cut-off values were considered in the correlation analysis.

The results shown in Fig. 3 will be conversed in the discussions section, but due to the number of features retained, 0.8 was considered as the optimal cut-off value for dimensionality reduction in this study. When the cavity and normal datasets were considered separately, the number of features retained with a 0.8 cut-off value in the two different consolidation measures were 11 and 12 in the SD measure and 12 and 12 in the variance measure, respectively. For both measures, only 10 features were common to the normal and cavity dataset (as seen in Fig. 3), with either 1 or 2 additional unique features retained. The features retained are recorded in Table 1 with the unique features emphasized in bold.

**Table 1:** Details of the features retained for the two different consolidation measures in the cavity and normal datasets, respectively, when a 0.8 cut-off value was used in the Pearson’s correlation analysis

Measure	Cavity CXR dataset	Normal CXR Dataset
SD	glcm_Correlation	glcm_Correlation
	gldm_DependenceEntropy	**glcm_DifferenceEntropy**
	gldm_DependenceNonUniformityNormalized	gldm_DependenceEntropy
	gldm_DependenceVariance	gldm_DependenceNonUniformityNormalized
	gldm_SmallDependenceLowGrayLevelEmphasis	gldm_DependenceVariance
	glrlm_RunEntropy	**gldm_LargeDependenceHighGrayLevelEmphasis**
	**glrlm_RunLengthNonUniformityNormalized**	gldm_SmallDependenceLowGrayLevelEmphasis
	glrlm_ShortRunLowGrayLevelEmphasis	glrlm_RunEntropy
	glszm_ZoneEntropy	glrlm_ShortRunLowGrayLevelEmphasis
	ngtdm_Busyness	glszm_ZoneEntropy
	ngtdm_Contrast	ngtdm_Busyness
		ngtdm_Contrast
Variance	glcm_Correlation	glcm_Correlation
	gldm_DependenceEntropy	glcm_DifferenceEntropy
	gldm_DependenceNonUniformityNormalized	**gldm_DependenceEntropy**
	gldm_DependenceVariance	gldm_DependenceNonUniformityNormalized
	gldm_SmallDependenceLowGrayLevelEmphasis	gldm_DependenceVariance
	**glrlm_RunEntropy**	**gldm_LargeDependenceHighGrayLevelEmphasis**
	**glrlm_RunLengthNonUniformityNormalized**	gldm_SmallDependenceLowGrayLevelEmphasis
	glrlm_ShortRunLowGrayLevelEmphasis	glrlm_ShortRunLowGrayLevelEmphasis
	glszm_GrayLevelNonUniformity	glszm_GrayLevelNonUniformity
	glszm_ZoneEntropy	glszm_ZoneEntropy
	ngtdm_Busyness	ngtdm_Busyness
	ngtdm_Contrast	ngtdm_Contrast

The features not common to both datasets are highlighted in bold

*glcm* gray level cooccurrence matrix, *gldm* gray level dependence matrix, *glrlm* gray level run length matrix, *glszm* gray level size zone matrix, *ngtdm* neighboring gray tone difference matrix

### Model results

The radiomic signatures obtained for both the SD and variance measures contained the same number of features (10), but the features included in their respective signatures differed (see Table 1). Two separate random forest models were therefore built, using a random walk oversampling technique, to model each of the two radiomic signatures, respectively. We note similar model performance for both models across all performance metrics (see Table 2).

**Table 2:** Performance comparison of the SD and variance radiomic signature-based models showing the AUC measures with the corresponding confidence intervals (95% CI), accuracy, sensitivity, specificity, and precision

	SD	Variance
AUC (95% CI)	0.9444 (0.8762; 0.9814)	0.9288 (0.9046; 0.9843)
Accuracy	0.8333	0.8750
Sensitivity	0.7708	0.8542
Specificity	0.8958	0.8958
Precision	0.8810	0.8913

## Discussion

The prompt diagnosis of PTB is vital for providing timely and accurate treatment, as a delay in treatment can lead to poor outcomes [17]. Biological methods are the gold standard for TB diagnosis, but culture or smear analysis takes time [30]. CXR are immediately available but cannot be used as a standalone tool for diagnosis. It has been shown that radiomics can improve discrimination performance and detection of medical images compared with those made by radiologists alone [9, 17, 18]. In this study, we developed a well performing radiomic model that could assist clinicians with the diagnosis of cavities due to TB on CXR. When added to the clinical signs and symptoms, this might reduce requirements for laboratory results and shorten time to treatment. It can also improve the accuracy of CXR reporting currently regaining prominence as a high-volume screening tool. The radiomic model that can detect cavities will also be useful in future PTB management studies when serial CXR, with their corresponding models, are studied.

For model construction, we first had to address the challenge of PTB disease segmentation that is required when quantifying X-rays using radiomic feature extraction. We developed a sliding window segmentation that allowed the extracted radiomic features to mimic the textural changes across the lung region caused by the disease. Inspiration for this newly developed segmentation method was obtained from a previous study that used deep learning approaches to sub-divide the lung region on a CXR into multiple stationary blocks [31]. They then used multi-instance learning (MIL) to classify each block as either a normal or a TB-manifestation class for TB diagnosis [31]. Instead of the stationary blocks, we introduced a sliding window approach to ensure that the boundaries between feature windows are well resolved.

An advantage of the secondary segmentation is that it eliminates the need to accurately delineate the diseased ROI which is time consuming and difficult in non-neoplastic diseases. It is also completely automated which eliminates observer-bias and increases reproducibility. The disadvantage of the secondary segmentation is that it increases the dimensionality of the data significantly, but this was addressed by introducing two different consolidation measures before performing traditional dimensionality reduction and model construction.

In previous studies on radiomic signature or nomogram construction for PTB, feature extraction was used to quantify digital medical images for the purpose of comparing, or differentiating, PTB from other pulmonary diseases, mainly tumors [13, 24, 32–34]. To the best of our knowledge, radiomics has not yet been used for the purpose of PTB diagnosis or disease management. The previously mentioned radiomics studies were performed from CT or PET/CT images [13, 24, 32–34], which seems redundant when patients in countries where PTB is most prevalent have very limited access to three-dimensional imaging modalities [35, 36]. For this reason, we used relevant 2D CXR for segmentation and feature extraction.

Although planar images are an unpopular modality for radiomic studies, a previous study was found where they applied a unique segmentation using a deep learning approach to train a model to automatically identify the thoracic disease in the lung and to generate bounding boxes around it [37]. In this study, radiomics features were used to create heat maps to assist the model in identifying the disease, rather than to quantify disease characteristics [37].

### Signature development

To develop a radiomic signature for PTB from CXR, dimensionality reduction was required to highlight the most important features and to remove redundant features. Pearson correlations analysis used in this study assumes that the data are normally distributed. We noted that most of the variables being analyzed are normally distributed and only a small proportion of the variables violate the normality assumption which was tested using the Shapiro–Wilk test. The correlation coefficients should therefore be largely unbiased and unaffected. Furthermore, various studies have indicated that Pearson correlations are robust to violations in the underlying assumptions [38], particularly when the normality assumption is violated. This then eliminated the consideration to use nonparametric alternatives to calculate the correlations between each feature given the few violations of the underlying normality assumption. Other less successful dimensionality reduction models considered for this study were: Inter-class correlation (ICC), Lasso regression, Factor analysis, Standardizing and Mean-absolute-deviation (MAD).

No recommendation on an optimal cut-off value for Pearson Correlations dimensionality reduction could be found in the literature. One study did, however, mention using 0.8, without validation [24]. We therefore evaluated three different cut-off values, 0.7, 0.8 and 0.9, in this study. When a cut-off value of 0.7 was used in the correlation analysis, it retained very few common features (3 and 5 features: 3.2 and 5.3%) in the different measures. This was found to be too conservative and eliminated some features that might be useful. A 0.9 cut-off value retained the most features (21 and 22 features; 22.5 and 23.6%), but this is too liberal and not useful in the context of dimension reduction. It was decided that 0.8 is therefore a balanced cut-off value to be applied.

Two general statistical methods were considered to quantify and consolidate the 3721 windows’ extracted features for each CXR. By statistical definition variance and standard deviation gives an indication of how much each data entry in a group differs from the mean of the group [23]. Average, median and IQR measures were also initially considered, but by definition they all average out the data and give no indication of the spread in the data [23]. From their statistical definition, these three related measures should produce results similar to when the secondary segmentation would have been disregarded. These three consolidation methods are therefore meaningless to achieve the study's aim to evaluate the spread in the radiomic features across the lung region and were ignored.

Two separate signatures for the SD and variance measures were developed by only including the features that were common to both the normal and cavity dataset for each measure, respectively. Each signature consists of 10 features, 9 common and 1 different feature (see Table 1). No first-order statistical features were included in this signature as these features use basic statistical algorithms to describe the value and distribution of the pixels in the ROI [15] and has no concern for spatial relationships [39]. Texture features are calculated by using the statistical inter-relationship between the pixels in the ROI [39].

### Model construction

The objective of model construction was to develop a noninvasive tool which can automatically differentiate cavities seen on CXR of people suffering from PTB and normal CXR to further assist with PTB diagnosis. As an additional benefit, a successful model will also prove the effectiveness and accuracy of the secondary segmentation introduced. The classification results (Table 2) of both models developed using the SD and variance radiomic signatures showed strong diagnostic power across most measures.

Theoretically, machine learning algorithms are most suitable for samples with uniform distributions in the model training process [40]. For this reason, the data were adjusted using the random walk oversampling technique which is an algorithm that generates synthetic instances so that the mean and SD of the numerical attributes remain close to the original data [27, 28]. This technique did correct the imbalance between the cavity and normal groups in the sampling distribution and therefore, improved the classification performance of the model. Four other sampling strategies were also considered but performed less convincingly: oversampling, synthetic minority sampling technique (SMOTE), simulation and majority weighted oversampling technique (MWMOT).

To construct the random forest models, a grid search cross-validation method was employed to determine the ideal number of variables to try at each tree split with 15 folds and 5 repeats to further limit overfitting. The grid search method is an exhaustive method commonly used to find the optimal parameter value by considering all possible combinations of these values for the model so that the classifier can more accurately predict the unlabelled or testing data [29]. Across both random forest models, the grid search cross-validation method indicated that the ideal number of variables to try at each tree split was 1.

**Fig. 4:**
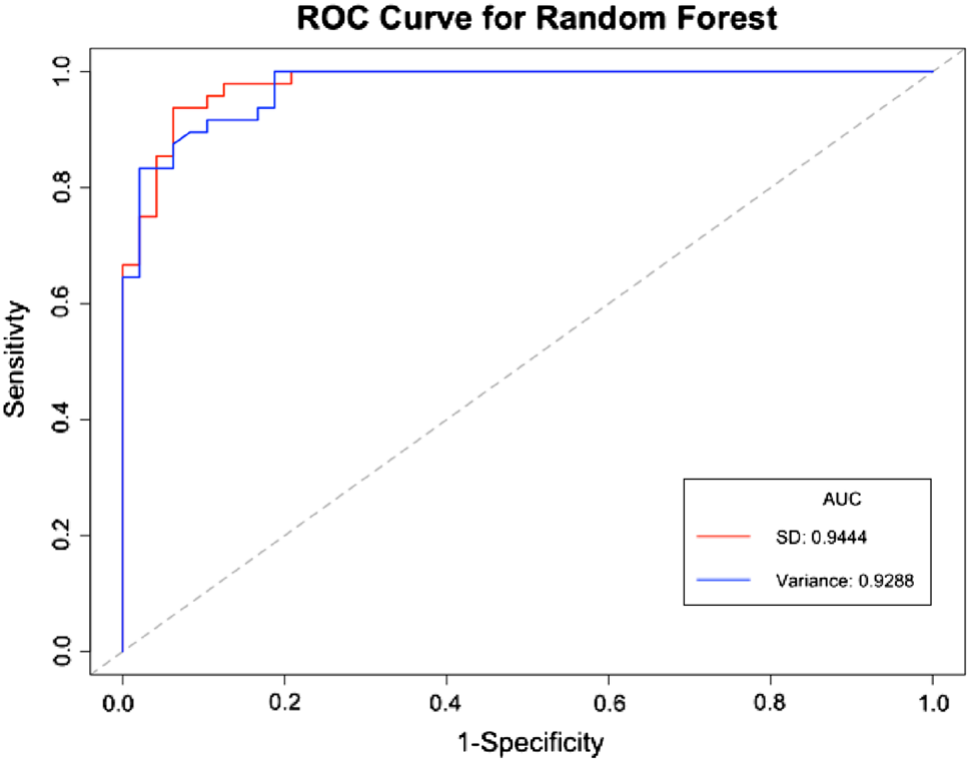
Receiver-operating characteristic (ROC) curves for the SD and variance signature-based random forest model using a random walk oversampling technique

As a result, the SD model performed marginally better than the variance model having a higher AUC value of 0.9444 (95% CI, 0.8762; 0.9814), which is larger than the variance model’s AUC value of 0.9288 (95% CI, 0.9046; 0.9843), but the 95% CI for the variance model is narrower which means there is less range in this estimate. This is visually supported by Fig. 4. The variance model had a better classification accuracy than the SD model with 87.50% and 83.33%, respectively, indicating that the variance model correctly predicted more cavities to the total observations in the data than the SD model. The variance model once again had a better model sensitivity than the SD model with a measure of 85.42% and 77.08%, respectively, which indicates that the variance model correctly identifies 85.42% of all cavity CXR. Interestingly, both models have a specificity measure of 89.58% which indicates that both models will identify 89.58% of patients who do not have cavities, i.e., have normal CXR. The variance model once again had a higher precision value than the SD model with a precision of 89.13 and 88.1%, respectively. This indicates that when the variance model predicts a cavity, it is correct 88.1% of the time.

Three other popular machine learning algorithms were also considered for model construction: Logistic regression and Lasso Regression (with a minimum error and one standard error away). All three performed poorly compared to the random forest model in correctly classifying the cavities due to the complex nature of the data and the ability of the random forest model to classify less distinctive groups with overlapping information for the classes.

Although we were able to develop robust radiomics models, there are limitations to this study. One major restriction is caused by the intrinsically superimposed nature of CXR images. Ribs and other higher density overlying structures cause noise in the lungs that is detected in the radiomic features. Currently, there are some attempts to develop bone suppression software that can retrospectively remove the ribs from CXR [41], but these models were not yet matured enough to apply to our unseen dataset. The successful removal of all superimposed high-density structures might further improve the performance of our model. Cavities are a single representation of PTB, but it is also a common challenge in image-based studies that multiple radiological TB expressions (e.g., adenopathy, infiltrates, and plural effusions) are present on a single X-ray. It is important to note that these other radiological expressions that might be present on the cavity CXR dataset can cause some subjectivity to the outcome of this study. Our sliding window segmentation method was only tested on a single representation of PTB. Future perspectives will extend this technique to other presentations and clinical models.

## Conclusion

In this study, two separate radiomic models were constructed, both of which achieved good classification accuracy for normal chest X-rays and cavities on X-rays of people suffering from pulmonary TB. This was achieved by the introduction of a secondary sliding window segmentation that was superimposed on a conventional automatic lung segmentation. This reproducible automatic segmentation method eliminates the difficult and labor-intensive manual disease delineation task, and it alleviates the subjectivity introduced by human judgement on X-rays. The well performing radiomic model could assist clinicians with the prompt diagnosis of pulmonary TB using digital chest X-rays. Accompanied with clinical signs and symptoms, it might aid diagnosis and commencement of pulmonary TB treatment and improve the accuracy of high-volume X-ray TB screening or surveillance programs.

## References

[1] WHO (2022) Global Tuberculosis Report 2022. ISBN 978-92-4-006172-9. World Health Organization, https://www.who.int/teams/global-tuberculosis-programme/tb-reports/global-tuberculosis-report-2022

[2] Chassagnon G, Vakalopoulou M, Paragios N, Revel MP (2020) Artificial intelligence applications for thoracic imaging. Euro J Radiol, 123. 10.1016/j.ejrad.2019.108774 10.1016/j.ejrad.2019.108774 31841881

[3] Karargyris A, Siegelman J, Tzortzis D, Jaeger S, Candemir S, Xue Z, Santosh KC, Vajda S, Antani S, Folio L, Thoma GR (2016). Combination of texture and shape features to detect pulmonary abnormalities in digital chest X-rays. Int J Comp Assist Radiol Surg J Interdisciplinary Res Development Applicat Image Guided Diagn Therapy.

[4] Tan JH, Acharya UR, Tan C, Abraham KT, Lim CM (2012). Computer-assisted diagnosis of tuberculosis: a first order statistical approach to chest radiograph. J Med Syst.

[5] Harisinghani MG, McLoud TC, Shepard J-AO, Ko JP, Shroff MM, Mueller PR (2000). Tuberculosis from head to toe. Radiographics.

[6] Stephen SFY, Hugo JWLA (2016). Applications and limitations of radiomics. Phys Med Biol.

[7] Brady A, Laoide RÓ, McCarthy P, McDermott R (2012). Discrepancy and error in radiology: concepts, causes and consequences. Ulst Med J.

[8] Kumar V, Gu Y, Basu S, Berglund A, Eschrich SA, Schabath MB, Forster K, Aerts HJ, Dekker A, Fenstermacher D, Goldgof DB, Hall LO, Lambin P, Balagurunathan Y, Gatenby RA, Gillies RJ (2012). Radiomics: the process and the challenges. Magn Reson Imaging.

[9] Gillies RJ, Kinahan PE, Hricak H (2016). Radiomics: images are more than pictures, they are data. Radiology.

[10] Avanzo M, Wei L, Stancanello J, Vallières M, Rao A, Morin O, Mattonen SA, El Naqa I (2020). Machine and deep learning methods for radiomics. Med Phys.

[11] Papanikolaou N, Matos C, Koh DM (2020). How to develop a meaningful radiomic signature for clinical use in oncologic patients. Cancer Imaging.

[12] Du Plessis T, Rae WID, Sathekge MM (2021). Pulmonary tuberculosis diagnosis, differentiation and disease management: a review of radiomics applications. Polish J Med Phys Eng.

[13] Bei W, Min L, He M, Fangfang H, Yan W, Shunying Z, Zhimin L, Tong Y, Jie T, Di D, Yun P (2019). Computed tomography-based predictive nomogram for differentiating primary progressive pulmonary tuberculosis from community-acquired pneumonia in children. BMC Med Imaging.

[14] van Timmeren JE, Cester D, Tanadini-Lang S, Alkadhi H, Baessler B (2020). Radiomics in medical imaging—“how-to” guide and critical reflection. Insights Imaging.

[15] Rizzo S, Botta F, Raimondi S, Origgi D, Fanciullo C, Morganti AG, Bellomi M (2018). Radiomics: the facts and the challenges of image analysis. Euro Radiol Experimental.

[16] Çallı E, Sogancioglu E, van Ginneken B, van Leeuwen KG, Murphy K (2021) Deep learning for chest X-ray analysis: a survey. Med Image Anal 72:102125 10.1016/j.media.2021.102125 10.1016/j.media.2021.102125 34171622

[17] Cui EN, Yu T, Shang S-J, Wang X-Y, Jin Y-L, Dong Y, Zhao H, Luo Y-H, Jiang X-R (2020) Radiomics model for distinguishing tuberculosis and lung cancer on computed tomography scans. World J Clin Cases 8 (21):5203–5212 10.12998/wjcc.v8.i21.5203 10.12998/wjcc.v8.i21.5203 PMC7674727 33269256

[18] Lohmann P, Bousabarah K, Hoevels M, Treuer H (2020). Radiomics in radiation oncology-basics, methods, and limitations. Strahlentherapie und Onkologie : Organ der Deutschen Rontgengesellschaft [et al].

[19] Skoura E, Zumla A, Bomanji J (2015). Imaging in tuberculosis. Int J Infect Dis.

[20] Scapicchio C, Gabelloni M, Barucci A, Cioni D, Saba L, Neri E (2021). A deep look into radiomics. Radiol Med (Torino).

[21] Zwanenburg A, Vallières M, Abdalah MA, Aerts HJWL, Andrearczyk V, Apte A, Ashrafinia S, Bakas S, Beukinga RJ, Boellaard R, Bogowicz M, Boldrini L, In B, Cook GJR, Davatzikos C, Depeursinge A, Desseroit MC, Dinapoli N, Dinh CV, Echegaray S (2020). The image biomarker standardization initiative: Standardized quantitative radiomics for high-throughput image-based phenotyping. Radiology.

[22] Dixon SAP (2019) Using deep learning to segment chest X-rays for the analysis of pneumonia. University of Sydney.

[23] Bain LJ, Engelhardt M (1992) Introduction to probability and mathematical statistics. The Duxbury advanced series in statistics and decision sciences, 2nd ed. edn. Duxbury Press, Belmont.

[24] Du D, Gu J, Chen X, Lv W, Feng Q, Rahmim A, Wu H, Lu L (2021) Integration of PET/CT radiomics and semantic features for differentiation between active pulmonary tuberculosis and lung cancer. Molecular Imaging Biol 23 (2). 10.1007/s11307-020-01550-4 10.1007/s11307-020-01550-4 33030709

[25] Devore JL, Berk KN (2012) Modern mathematical statistics with applications. Springer texts in statistics, 2nd edn. Springer, New York. 10.1007/978-1-4614-0391-3

[26] Hastie T, Tibshirani R, Friedman JH (2009) The elements of statistical learning : data mining, inference, and prediction. Springer series in statistics, 0172–7397, 2nd edn. Springer, New York

[27] Cordón I, García S, Fernández A, Herrera F (2018). Imbalance: oversampling algorithms for imbalanced classification in R. Knowl-Based Syst.

[28] Zhang H, Li M (2014). RWO-Sampling: a random walk over-sampling approach to imbalanced data classification. Information Fusion.

[29] Ramadhan MM, Sitanggang IS, Nasution FR, Ghifari A (2017) Parameter tuning in random forest based on grid search method for gender classification based on voice frequency. DEStech Trans Compu Sci Eng (CECE). 10.12783/dtcse/cece2017/14611

[30] Che-Engku-Chik CEN, Yusof NA, Abdullah J, Othman SS, Mat Said MH, Wasoh H (2016). Detection of tuberculosis (TB) using gold standard method, direct sputum smears microscopy, PCR, qPCR and electrochemical DNA sensor: a mini review.

[31] Khatibi T, Shahsavari A, Farahani A (2021). Proposing a novel multi-instance learning model for tuberculosis recognition from chest X-ray images based on CNNs, complex networks and stacked ensemble. Phys Eng Sci Med.

[32] Shi W, Zhou L, Peng X, Ren H, Wang Q, Shan F, Zhang Z, Liu L, Shi Y (2019) HIV-infected patients with opportunistic pulmonary infections misdiagnosed as lung cancers: the clinicoradiologic features and initial application of CT radiomics. J Thoracic Dis 11 (6):2274–2286. 10.21037/jtd.2019.06.22 10.21037/jtd.2019.06.22 PMC6626777 31372264

[33] Feng B, Chen X, Chen Y, Liu K, Li K, Liu X, Yao N, Li Z, Li R, Zhang C, Ji J, Long W (2020) Radiomics nomogram for preoperative differentiation of lung tuberculoma from adenocarcinoma in solitary pulmonary solid nodule. Euro J Radiol 128 10.1016/j.ejrad.2020.109022 10.1016/j.ejrad.2020.109022 32371184

[34] Cui EN, Yu T, Shang SJ, Wang XY, Jin YL, Dong Y, Zhao H, Luo YH, Jiang XR (2020) Radiomics model for distinguishing tuberculosis and lung cancer on computed tomography scans. World J Clin Cases 8 (21):5203–5212. 10.12998/wjcc.v8.i21.5203 10.12998/wjcc.v8.i21.5203 PMC7674727 33269256

[35] Melendez J, Bv G, Maduskar P, Philipsen RHHM, Ayles H, Sánchez CI (2016). On combining multiple-instance learning and active learning for computer-aided detection of tuberculosis. IEEE Trans Med Imaging.

[36] Santosh KC, Antani S (2018). Automated chest X-ray screening: can lung region symmetry help detect pulmonary abnormalities?. IEEE Trans Med Imaging.

[37] Han Y, Chen C, Tang L, Lin M, Jaiswal A, Ding Y, Peng Y (2021) Using radiomics as prior knowledge for abnormality classification and localization in chest X-rays. AMIA Annu Symp Proc 21:546–555 PMC8861661 35308939

[38] Havlicek LL, Peterson NL (1976) Robustness of the Pearson correlation against violations of assumptions. Perceptual Motor Skills 43 (3_suppl):1319–1334 10.2466/pms.1976.43.3f.1319

[39] van Griethuysen JJM, Fedorov A, Parmar C, Hosny A, Aucoin N, Narayan V, Beets-Tan RGH, Fillion-Robin JC, Pieper S, Aerts H (2017). Computational radiomics system to decode the radiographic phenotype. Can Res.

[40] Wang X-H, Long L-H, Cui Y, Jia AY, Zhu X-G, Wang H-Z, Wang Z-H, Wang W-H (2019) A MRI-based radiomics model for preoperative prediction of five-year survival status in hepatocellular carcinoma. J Clin Oncol 37 (15_suppl):e15596 10.1200/JCO.2019.37.15_suppl.e15596

[41] Zhou Z, Zhou L, Shen K (2020). Dilated conditional GAN for bone suppression in chest radiographs with enforced semantic features. Med Phys.

